# Structural and compositional analysis of (InGa)(AsSb)/GaAs/GaP Stranski–Krastanov quantum dots

**DOI:** 10.1038/s41377-021-00564-z

**Published:** 2021-06-15

**Authors:** Raja S. R. Gajjela, Arthur L. Hendriks, James O. Douglas, Elisa M. Sala, Petr Steindl, Petr Klenovský, Paul A. J. Bagot, Michael P. Moody, Dieter Bimberg, Paul M. Koenraad

**Affiliations:** 1 Department of Applied Physics, Eindhoven University of Technology, 5612 AZ Eindhoven, The Netherlands; 2 Department of Materials, University of Oxford, Parks Road, Oxford, OX1 3PH UK; 3 Center for Nanophotonics, Institute for Solid State Physics, TechnischeUniversität Berlin, Hardenbergstr. 36, 10623 Berlin, Germany; 4 EPSRC National Epitaxy Facility, The University of Sheffield, North Campus, Broad Lane, Sheffield, S3 7HQ UK; 5 Department of Condensed Matter Physics, Faculty of Science, Masaryk University, Kotlářská267/2, 61137 Brno, Czech Republic; 6 Huygens-Kamerlingh Onnes Laboratory, Leiden University, P.O. Box 9504 2300 RA Leiden, Netherlands; 7 Czech Metrology Institute, Okružní 31, 63800 Brno, Czech Republic; 8 “Bimberg Chinese-German Center for Green Photonics” Changchun Institute of Optics, Fine Mechanics and Physics, Chinese Academy of Sciences at CIOMP, 13033 Changchun, China

**Keywords:** Photonic devices, Quantum dots

## Abstract

We investigated metal-organic vapor phase epitaxy grown (InGa)(AsSb)/GaAs/GaP Stranski–Krastanov quantum dots (QDs) with potential applications in QD-Flash memories by cross-sectional scanning tunneling microscopy (X-STM) and atom probe tomography (APT). The combination of X-STM and APT is a very powerful approach to study semiconductor heterostructures with atomic resolution, which provides detailed structural and compositional information on the system. The rather small QDs are found to be of truncated pyramid shape with a very small top facet and occur in our sample with a very high density of ∼4 × 10^11^ cm^−2^. APT experiments revealed that the QDs are GaAs rich with smaller amounts of In and Sb. Finite element (FE) simulations are performed using structural data from X-STM to calculate the lattice constant and the outward relaxation of the cleaved surface. The composition of the QDs is estimated by combining the results from X-STM and the FE simulations, yielding ∼In_*x*_Ga_1 − *x*_As_1 − *y*_Sb_*y*_, where *x* = 0.25–0.30 and *y* = 0.10–0.15. Noticeably, the reported composition is in good agreement with the experimental results obtained by APT, previous optical, electrical, and theoretical analysis carried out on this material system. This confirms that the InGaSb and GaAs layers involved in the QD formation have strongly intermixed. A detailed analysis of the QD capping layer shows the segregation of Sb and In from the QD layer, where both APT and X-STM show that the Sb mainly resides outside the QDs proving that Sb has mainly acted as a surfactant during the dot formation. Our structural and compositional analysis provides a valuable insight into this novel QD system and a path for further growth optimization to improve the storage time of the QD-Flash memory devices.

## Graphical abstract

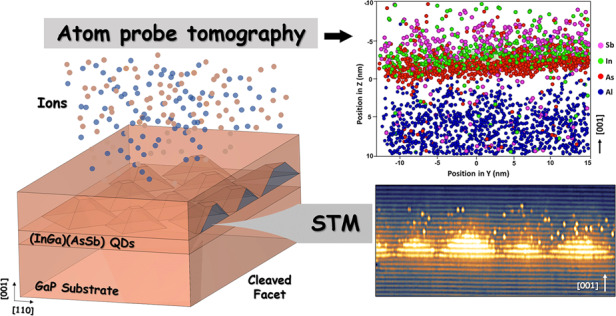

## Introduction

Optoelectronic devices with self-assembled quantum dots (QDs) as an active medium have shown superior properties in many applications, such as semiconductor lasers^1,2^, single and entangled photon emitters^3–12^, solar cells^13^, and quantum information technology^14–19^. Antimony-based type-II QDs^20–25^ that exhibit hole confinement are especially suitable for memory applications: the so-called QD-Flash memories^25–27^. The higher effective mass of holes leads to a higher localization energy and storage time compared to type-I QDs based on electron confinement^28,29^. QD-Flash is a relatively new technology, it is proposed as a nonvolatile memory system with nanosecond read/write/erase times, and endurance of 10^15^ read/write cycles^30,31^.

The write time of the QD-Flash device is limited only by the thermal capture of charge carriers into the QDs, which occurs on the order of picoseconds at room temperature. The erase time strongly depends on the localization energy and the applied bias. The storage time “*τ*” mainly depends on localization energy (depth of the localization potential) and the capture cross-section (*σ*_∞_-scattering probability of holes)^30–32^. High localization energies with low-capture cross-section are preferred to obtain long storage time. Both parameters are strongly influenced by the size, shape, and composition of the QDs, as the bigger dots increase the localization potential at the expense of increasing capture cross-section^33^. Careful tuning of these parameters would allow the fabrication of QD-Flash devices with longer storage times. The use of GaP as a matrix material for III–V semiconductors has attracted much research interest due to a small lattice mismatch of 0.4% with Si, which results in defect-free growth when grown on Si substrates^34,35^. This would allow easy integration of QD-based devices with the well-established Si technology.

A localization time of 10^6^ years was proposed by Marent et al. for GaSb/AlAs QDs^36^, but at least 10 years is necessary to reach a practical nonvolatile system. The GaSb/GaP QDs with hole confinement grown by molecular beam epitaxy (MBE) have shown the longest storage time of 4 days^37^. Instead, for QDs grown via metal-organic vapor phase epitaxy (MOVPE), the current storage time for pure In_0.5_Ga_0.5_As/GaP QDs is 230 s at room temperature^38^, while an improvement of one order of magnitude was obtained by adding Sb during the QD growth, leading to a record storage time of 1 h at room temperature, as reported by Sala et al.^27,39^. A complete growth optimization of the (InGa)(AsSb)/GaAs/GaP QDs was reported by Sala et al.^26^ and a detailed theoretical analysis of the quaternary QD system was published by P. Klenovský et al.^32^. The storage time of these QDs was measured utilizing deep-level transient spectroscopy^27^. The optical transitions of the similar QDs were studied through excitation and temperature-dependent photoluminescence (PL) published by Steindl et al.^40^.

Cross-sectional scanning tunneling microscopy (X-STM) is capable of resolving semiconductor nanostructures with atomic resolution. The precise structural data and fundamental understanding of the growth mechanism are necessary to optimize QDs for various optoelectronic applications. X-STM can provide the size, shape, and composition of the embedded QDs. The structural and compositional changes after overgrowth, such as intermixing, segregation, and morphological changes in QDs can also be studied by X-STM^41–55^. Information about confined states in QDs can be obtained by scanning tunneling spectroscopy^56–58^. Furthermore, X-STM can also resolve the effects of isoelectronic impurities in semiconductors (such as GaAs and InP) with atomic resolution^59–64^. Atom probe tomography (APT) has the potential to obtain the complete three-dimensional reconstruction of the topography along with mass spectral analysis, identifying, thus, different chemical species. Hence, it can provide detailed composition analysis of semiconductor nanostructures, such as QDs^48,65,66^.

In the present study, we investigate the (InGa)(AsSb)/GaAs/GaP QDs by X-STM to precisely determine the size, shape, composition, and density of the QDs with atomic resolution. Detailed composition analysis of the QDs by APT is also presented, further strengthening our results. Finite element (FE) simulations based on continuum elasticity theory are performed, in order to fit the experimental lattice constant and outward relaxation of the cleaved QD. Both X-STM results and FE simulations are used in conjunction to provide an estimation of the QD composition. The estimated composition is in good agreement with the composition analysis obtained from APT and the previous optical, electrical, and theoretical studies^27,32,40,67^. In addition, we report a detailed analysis of the segregation of constituent atoms from the QD layer into the capping layer, thus confirming the presence of Sb in the QD region, and providing valuable feedback for further growth optimization. We note that the sample, investigated here with X-STM and APT, is exactly the same as the one with a storage time record of 1 h at room temperature, measured via deep-level transient spectroscopy^27^.

## Results

The X-STM and APT results are presented in three sections. “QDs: size and shape” section describes the size and shape of QDs derived from many filled-state topographic X-STM images. The compositional analysis of QDs by APT is given in “QDs: composition” section, which is further supported by the FE simulations performed to fit the local lattice constant and outward relaxation of the cleaved QD. In “QDs: capping layer” section, we present a detailed analysis on the capping layer composition, where segregation of elements (Sb, In) from the QD layer into the capping layer is investigated.

### QDs: size and shape

**Fig. 1:**
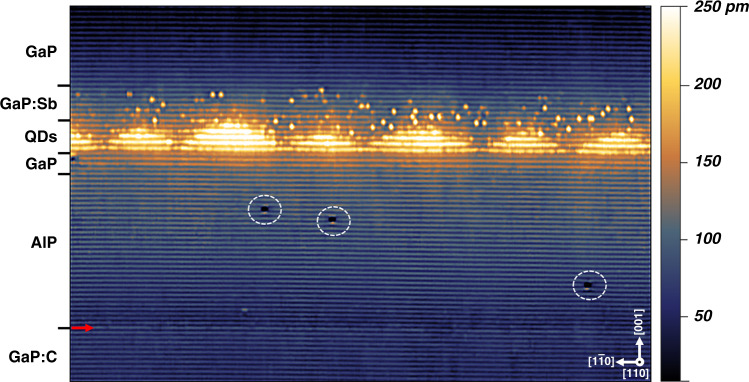
A 60 × 40 nm^2^ filled-state topographic image showing six full QDs taken at a bias voltage: *V*_b_ = −3.3 V and a tunnel current: *I*_t_ = 50 pA. The red arrow indicates the interface between GaP:C and AlP; the white circles indicate “P” vacancies; the dark to bright contrast (250 pm) in the image represents the relative height of the STM tip from the surface as shown to the right of the image in the color bar. The white arrow indicates the growth direction [001]

The growth started with a GaP buffer layer followed by 20 nm of AlP barrier layer to increase the hole localization energy. After capping the AlP with 2 nm of GaP, 5 monolayers (MLs) of GaAs interlayer (IL) was deposited to facilitate the Stranski–Krastanov (SK) growth of the QDs. Later, In_0.5_Ga_0.5_Sb was supplied to form QDs followed by a GaP capping layer. A detailed growth description and sample structure are provided in “Materials and methods” section. Fig. 1 shows a filled-state topographic image taken at a bias voltage (*V*_b_) = −3.3 V and a tunnel current (*I*_t_) = 50 pA. The brightness in the image marks the relative height of the STM tip above the surface. Multiple layers can be identified by brightness variations, resulting from the outward relaxation of the compressively strained layers due to the lattice mismatch (structural contrast). Note that the electronic contrast was suppressed by measuring at high negative bias voltages. At the bottom of Fig. 1, a sharp interface between GaP:C and AlP can be identified (indicated by a red arrow). The GaP and AlP have similar lattice constant leading to strain-free growth. The sharpness of the interface reflects the high quality of the growth. The three dark spots in the AlP region of Fig. 1 are “P” vacancies (white circles) created by Langmuir evaporation^68^ due to cleaving. The thickness of the AlP region measured by X-STM is 18 ± 0.5 nm and the moderately bright 3–4 bilayers (BLs) just below the QDs corresponds to the 2 nm GaP grown above the AlP. Since we do not observe the deposited GaAs IL, we suppose that most of the GaAs IL, deposited in order to facilitate the SK growth, is intermixed with the In_0.5_Ga_0.5_Sb supplied for QD formation, thus, creating a quaternary system, i.e., (InGa)(AsSb) QDs, as previously predicted^39,40^.

The QDs appear to be small in size and homogeneous in composition compared to conventional SKQDs^50^. Also, our QDs appear to be smaller than the InGaAs/GaAs/GaP QDs studied via X-STM by Prohl et al.^69^. This is in accordance with the atomic force microscopy (AFM) measurements on uncapped (InGa)(AsSb)/GaAs/GaP QDs^26,27,39^. The composition of the QDs is uniform in the center, but we observed some intermixing close to the edges of the QDs, since we see contrast fluctuations at the QD edges in X-STM images. As mentioned before, most of the GaAs IL is consumed during the QD formation, possibly the core of the QD is rich in GaAs with In and Sb incorporated close to the edges. Because the Sb acts as a surfactant^70^ and, thus, we expect higher In incorporation than Sb in these QDs. The surfactant effect of Sb assists in forming smaller QDs with higher density by reducing the adatom mobility on the growth surface^70,71^.

Our QDs have a near triangular shape in the cross-section images, suggesting a cleaving plane parallel to the diagonal of the pyramid. However, few QDs have a trapezoidal shape in the cross-section with a small top facet indicating a truncated pyramid shape in three dimensions. We suppose that cleaving through the center of the QD revealed a trapezoidal shape and away from the QD center revealed a triangular shape in the X-STM images. As expected from the capping procedure, the apex of the pyramid was dissolved during the overgrowth, slightly transforming the QDs shape. This process is commonly observed during the overgrowth of SKQDs^41,72–74^. It is important to point out that the density of the investigated QDs is as high as 4 × 10^11^ cm^−2^, which is very high compared to conventional SKQDs and twice that of the InGaAs/GaAs/GaP QDs^69^. The thickness of GaAs IL and the amount of deposited material strongly influence the QDs density and size. Due to the very high dot density, most of the QDs almost touch each other and share 1–2 MLs at the bottom, which can be clearly seen in Fig. 1. The effect of GaAs IL thickness on QD density was reported by Sala et al.^26,39^, where a maximum of 2 × 10^11^ cm^−2^ was observed for 6 MLs GaAs IL and the effect of other growth conditions, such as temperature, growth interruption (GRI) time, and Sb-flush was also studied. As already discussed above, the use of an Sb-flush, in which Sb acts as a surfactant, strongly affects the QD formation, by modifying the adatom surface diffusion, also is known for other III–V systems^71^. The effect of Sb can be clearly seen by comparing our results with InGaAs/GaAs/GaP QDs, where bigger QDs with a lower density were observed^69^. Fig. 1 shows a filled-state image where only group V elements are visible, the Sb segregation from the QD layer into the capping layer (both on and below the cleaved surface) can be identified just above the QDs. The brightest being the surface Sb atoms and the brightness decreases with increasing depth from the surface^75^.

**Fig. 2:**
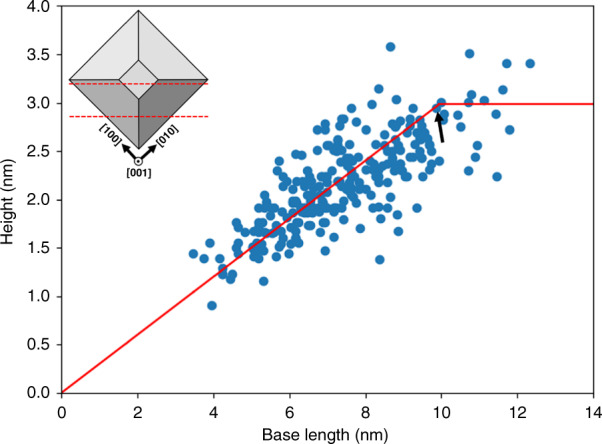
Height vs base length distribution of 261 individual QDs measured from X-STM images. The red line is a linear fit to the experimental data (blue). The black arrow indicates the position where the shape of the QDs changes from near triangle to trapezium, with a small top facet. On the left corner, the top view of the most probable QD model is given with dotted red lines, indicating the cleaving planes at two different positions

**Fig. 3:**
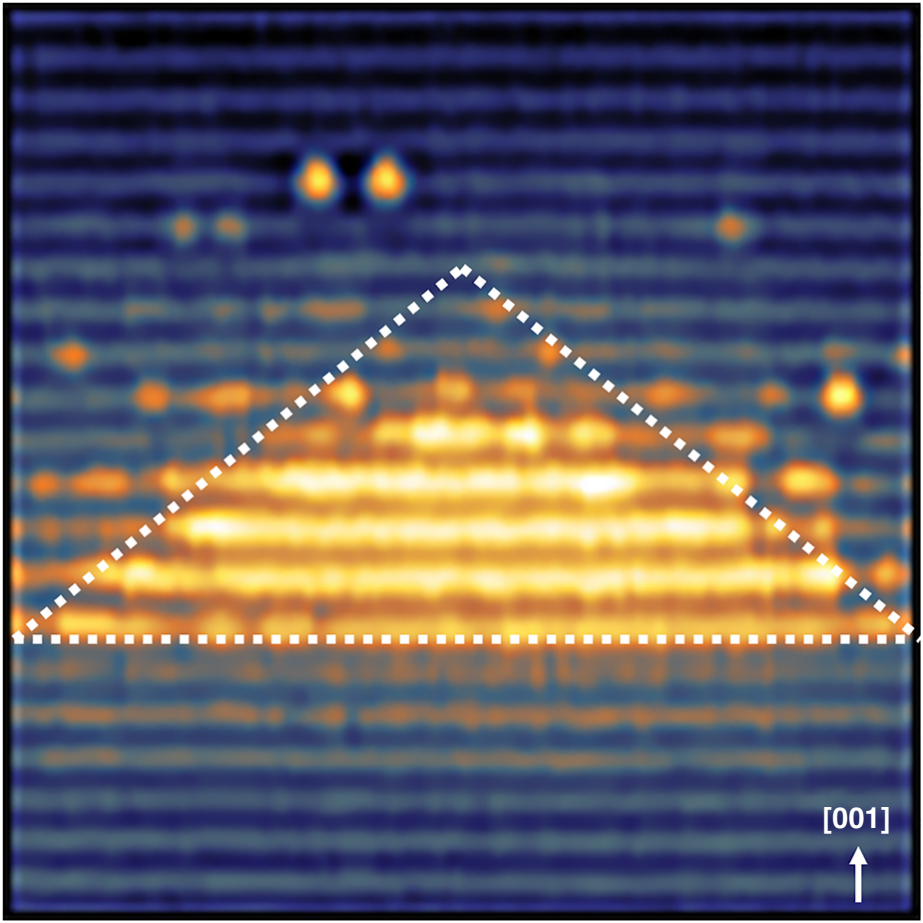
A 11 × 11 nm^2^ filled-state image showing one of the biggest QD taken at *V*_b_ = −3.3 V and *I*_*t*_ = 50 pA with white dotted lines showing a cut through the pyramid. The diagonal base length of the QD is 11 ± 0.2 nm with a height of 5 BLs and the arrow indicates the growth direction [001]

The cleaving of the sample is arbitrary and need not be through the center of every dot, for this reason, the height and base length of 261 individual QDs are measured and plotted in Fig. 2. The height and base length are a function of cleaving position and are used to determine the orientation of the QDs, with respect to the cleaving plane by a simple geometrical model reported by Bruls et al.^76,77^. From Fig. 2, we find that there is a linear relationship between the height and base length of QDs, and the height of the QDs is saturated at 3.0–3.5 nm. This suggests that the cleaving is parallel to the diagonal of the truncated pyramid (model 2 from Bruls et al.^76^). It can be considered that the maximum base length is observed when the cleaving is through the center of the QD. So, the average base length at maximum height is 12 ± 0.8 nm, which is the diagonal of the square base model 2 from Bruls et al.^76^. The actual base length is $\sqrt 2$ times smaller, i.e., 8.5 ± 0.6 nm at a maximum height of 10 MLs, (i.e., 3.0 ± 0.4 nm), resulting in an aspect ratio (height to base length) of 0.25–0.35. Our analysis suggests very little inhomogeneity in the QDs size distribution. Prohl et al.^69^ reported an average base length of 12 nm and a height of 10 MLs for InGaAs/GaAs/GaP QDs. We observed a smaller base length and similar height for our Sb-based QDs, clearly showing the effect of Sb on QDs formation. One of the biggest QD found during the measurement is shown in Fig. 3, with white dotted lines indicating a cut through the pyramid. The dissolution of the QD apex (~2 BLs) can be observed in Fig. 3, thus forming a trapezoidal shape in the X-STM image.

### QDs: composition

**Fig. 4:**
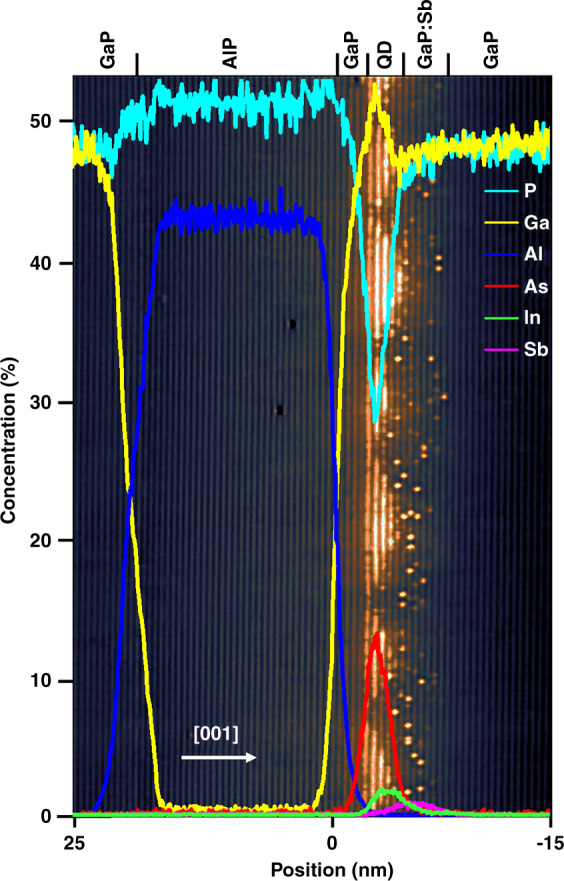
The APT concentration profiles of all the constituent elements (P, Ga, Al, As, In, and Sb) overlaid on top a 40 × 60 nm^2^ topographic filled-state X-STM image for better visualization and comparison. The apparent non-stoichiometry of the AlP layer is due to the unresolvable identification issue of complex phosphorus species in the 31 Da peak, as described in “QDs: composition” section. The arrow indicates the growth direction [001] and the different regions are indicated on top of the image

**Fig. 5:**
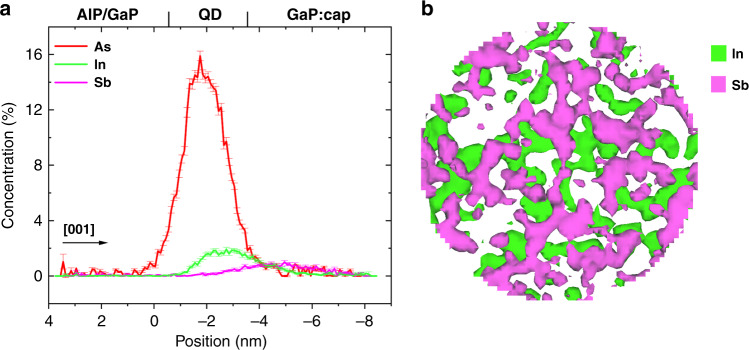
APT evaluation of the QD constituent elements. **a** APT concentration profile showing only As, In, and Sb is generated from the 30 nm diameter slice shown in panel **b** rather than from the full dataset that is shown in Fig. 4. The arrow indicates the growth direction [001]; **b** 30 nm diameter slice of iso-concentration surfaces created with In = 2.0 at.% and Sb = 1.0 at.%, showing the anticorrelation in the spatial distribution of the In- and Sb-rich regions

As mentioned in the growth description, 0.51 ML of In_0.5_Ga_0.5_Sb is deposited on top of the 5 MLs of GaAs IL in order to induce the QD formation. We will now estimate the indium concentration in the QDs assuming that all indium is incorporated in the QDs. It is easy to show that 0.51 ML of In_0.5_Ga_0.5_Sb corresponds to an estimated surface atomic density of indium atoms of 6.45 × 10^13^ cm^−2^. Considering a QD density of 2 × 10^11^ cm^−2^ as determined from AFM^26,27^, and in agreement with the density estimate that we made from the X-STM data, we conclude that about ∼323 In atoms are present in each QD. From the structural analysis, we know the base length (∼8.5 nm) and height (∼3 nm) of the pyramidal-shaped QDs and, thus, we can calculate the volume of each QD. This volume corresponds with ∼1164 group III positions per dot and thus an average In fraction of about ∼28% inside the QDs. There might be some unincorporated GaAs left at the bottom of the QDs acting as a sort of wetting layer. From the APT profiles shown in Figs. 4 and 5a, we can see that also some Sb, yet at a lower concentration than In, can be present in QDs.

Detailed compositional analysis of the QDs was performed by APT. An iso-concentration surface of 15% Al was created (labeled as 0 nm), and a proximity histogram^78^ with 0.1 nm bins was used to generate concentration profiles for the elements detected in the sample and part of it is shown in Fig. 4 (full concentration profile is shown in Fig. S6 of section S-3 in the Supplemental Material). Ionic species relating to Ga, P, As, Al, In, and Sb were all detected and identified using known isotopic masses and abundances. No mass peak overlaps were detected for In and Sb species and the majority of these were found in the In^+^ and Sb^2+^ states. The elemental concentration was calculated by decomposing and summing all contributing single and complex ionic species^79^. Mass spectra peak overlaps can occur that cannot be solved through standard methods using known isotopic abundances. This is particularly an issue for phosphorus as it is both monoisotopic and also readily forms complex ions, thus forming unsolvable mass spectra overlaps^80^ of the form ^31^${\mathrm{P}}_{\mathrm{X}}^ +$ and ^31^${\mathrm{P}}_{2{\mathrm{X}}}^{2 + }$ where the choice of ionic identity can double or half the phosphorus contribution. The apparent non-stoichiometry of the AlP layer in Fig. 4 is due to the above-mentioned ambiguity in the identification of the complex phosphorous species. The issue is made more challenging as the overlapped peak will in fact contain a ratio of each of the possible ions and so the exact contribution to the composition cannot be directly measured using current APT detection methods. This issue can also affect arsenic, as it is also monoisotopic and thus in this sample, the phosphorus contribution from the 31 Da (mass to charge-state ratio) peak can be from either $^{31}{\mathrm{P}}^ +$ or $^{31}{\mathrm{P}}_2^{2 + }$, with the preference for the former when the tip apex undergoes a higher electric field (such as when analyzing material that requires a higher evaporation field, such as AlP, or when the tip becomes progressively blunter during analysis) and the latter when tip apex is subject to a lower electric field^81^ (such as when analyzing through the AlP layer and into the underlying GaP layer). The voltage applied to the atom probe specimen is changed automatically to maintain a specific detection rate and so identity of ions within the 31 Da peak can shift between $^{31}{\mathrm{P}}_2^{2 + }$ and $^{31}{\mathrm{P}}_2^{2 + }$ during analysis, hence, giving apparent variation in composition shown in Fig. 4, where the 31 Da peak has been labeled as $^{31}{\mathrm{P}}_2^{2 + }$. While the proportion of ions labeled as either $^{31}{\mathrm{P}}^ +$ or $^{31}{\mathrm{P}}_2^{2 + }$ within the 31 Da peak can be imposed to produce a known stoichiometry in bulk regions with constant voltages, this does not hold for the changing voltages and resultant electric fields when analyzing through interfaces, which can also have a variable composition, and so this has not been carried out in order to prevent biasing the data. Although the exact composition of the QDs may not be readily obtained due to demanding analysis (especially at interfaces) of phosphorous-rich materials, the spatial and compositional trends in the non-phosphorus species can provide a great deal of information.

**Fig. 6:**
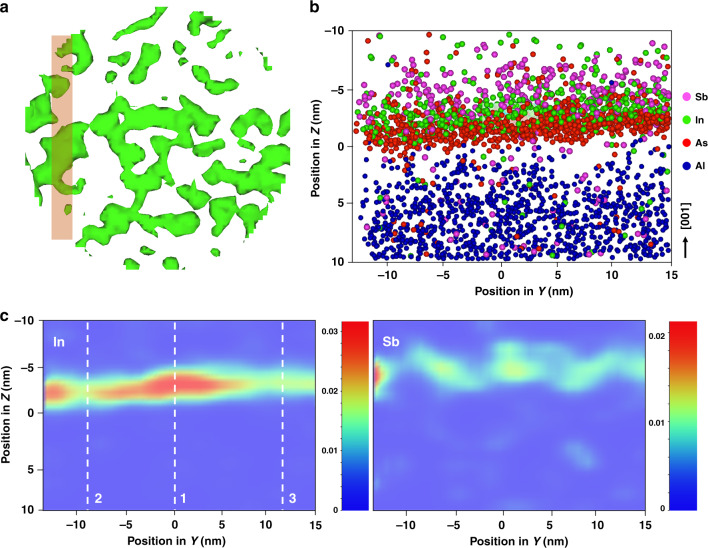
APT investigation of the QD region. **a** A 30 nm diameter slice of the iso-concentration surface created with In = 2.0 at.%, showing the In-rich areas, the shaded region represents a 5-nm thick cross-sectional slice taken to plot concentration maps of the elements; **b** cross-sectional atom map of the shaded region from panel **a**, showing the distribution of constituent elements (Al, As, In, and Sb); **c** cross-sectional concentration maps of In and Sb, the dotted lines indicate: (1) In-rich region, (2) In-poor region, and (3) In-poor but As-rich region. The exact composition as reported in the color bars is not completely quantitative due to the spatial blurring from trajectory aberrations that occurs during atom probe, where matrix material from the vicinity of the QDs can appear to be from inside the QDs

For a detailed analysis, a 60 nm diameter and 20 nm thick cylinder was extracted from the interface region containing the QD structures, an iso-concentration surface of 15% Al was created and a proximity histogram is used to generate concentration profiles of the elements involved in the QD multilayer system. This volume is seen overlaid on the X-STM image in Fig. 4 and can be expected to contain multiple QDs; therefore, concentration profiles will be an averaging of these dots and the intervening material. Interfacial widths on the order of a nanometer can be seen at the interface between the layers of material with different compositions and thus evaporation field requirements, this additional spatial blurring is common during the analysis of complex systems^82^. Clear peaks of As, In, and Sb can be observed in that order from the growth direction in Fig. 5a; however, the peak maxima are distinctly overset from each other, showing a degree of intermixing between the layers. The overlap between the As and In layers is likely the result of GaAs IL consumption during QD formation. The In profile shows a sharper increase from the growth direction and a more gradual decline into the Sb profile, which is shorter and wider than that of the In profile. The As profile encompasses the majority of the In profile and also shows a gradual decline into the Sb profile. The gradual change of slope in concentration profile along the growth direction indicates the segregation of elements into the capping layer, confirmed by the X-STM analysis. The thickness of the In layer, as measured from half peak maxima, is ~3.0 nm and is in line with the measured height of the QDs.

In order to more precisely analyze the QD structures, iso-concentration surfaces of In and Sb were generated from the cylindrical volume and shown top-down facing the growth direction in Fig. 5b. These show that the planar distribution of In and Sb appears to anticorrelated to some degree, which is in agreement with the X-STM observations that the In is likely contained within the QDs themselves, while the Sb acts as a surface coating layer. The spatial trend of the concentration profiles clearly indicates a 2:1 ratio of In:Sb in the QD regions. The level of intermixing between the species that could plausibly be contained within the QDs, combined with the inherent spatial blurring from laser pulsed atom probe of complex multilayers, make the precise delineation and, thus, compositional and structural quantification of the QDs quite challenging. Therefore, to determine the height and width of QDs, we use the approach adapted from ref. ^66^. QDs are capped with a layer of GaP and, as stated earlier, truly quantitative concentrations of these P containing materials is challenging due to the self-overlap issue of complex phosphorus ions. However, it is still possible to compare relative concentrations of Sb and In within and between the QDs.

A 5 nm thick cross-section was extracted from this cylindrical volume, bisecting a region of high indium concentration as shown in Fig. 6a, the shaded region. A cross-sectional atom map of that region can be seen in Fig. 6b, revealing the layering of the various species. Concentration maps for this cross-section (shaded region) are plotted for In and Sb as shown in Fig. 6c. The exact composition as reported in the color bars is not completely quantitative due to the spatial blurring from trajectory aberrations that occurs during atom probe, where matrix material from the vicinity of the QDs can appear as a constituent material of the QD itself. There is a single In-rich region in the center indicated with dotted line 1, an In-poor region to the left (dotted line 2), and an As-rich but In-poor region to the right (dotted line 3) in Fig. 6c. A flattop triangular structure, similar to that shown in Fig. 3 can be seen in Fig. 6c in the In concentration map. This is ~3.0 nm in height and 14.0 nm in width agreeing well with the X-STM measurements. In Fig. 6b, the As layer appears to lay slightly below that of the In layer, but their concentration maxima are linked, this would agree with the observation that some of the GaAs IL still be present, but with the As atoms closer to the QD formation region being incorporated into the QDs. The most interesting part is the distribution of Sb, which can be clearly seen to be above the In-rich regions, as expected from the X-STM images and the larger scale APT concentration profiles, but also angled such that they are coating the edges of the In-rich triangular feature. 5 × 5 × 20 nm^3^ cuboid regions were placed over the In-rich (dotted line 1), In-poor (dotted line 2), and As-rich but In-poor (dotted line 3) regions within the cross-section in Fig. 6c. The In poor can be considered to be mainly between QDs, the In-rich region to be straight through QD and the As-rich but In-poor region is likely bisecting the edge of QD. The ratios of In:Sb were found to be ~1.8, 0.9, and 1.6 respectively, suggesting that there is a layer of both In and Sb between QDs, but that unincorporated Sb is also present on the edges and tops of the QDs.

**Fig. 7:**
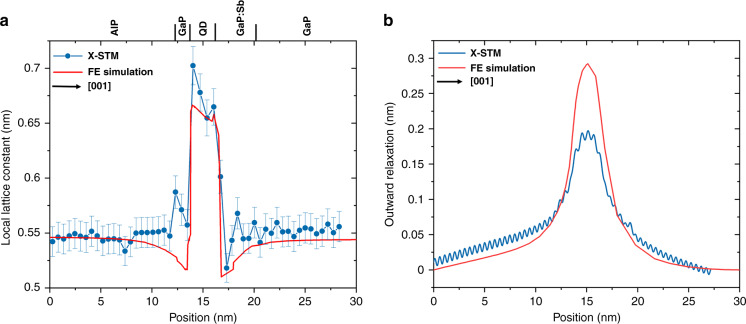
FE simulations fitting the experimental X-STM results. **a** Lattice constant profile of the biggest QD in Fig. 1 (second QD from left) as a function of position in the growth direction (from left to right). The blue points represent the measured lattice constant with an FE simulation in red for a composition of In0.3Ga0.7As0.85Sb0.15. Different layers are marked on top of the profile; **b** X-STM height profile showing the outward relaxation of the same cleaved QD with an FE simulation in red

The QD composition can also be estimated by determining the local lattice constant and the outward relaxation profile of the QDs after cleaving in combination with FE simulations. Figure 7a shows a lattice constant profile of the biggest QD in Fig. 1 (second QD from left), as a function of position in the growth direction. AlP and GaP have similar lattice constant that is reproduced in the measured lattice constant profile. The slight increase in lattice constant above the AlP layer is attributed to the interface between AlP and 2 nm GaP. We observed a similar kink in the lattice constant profile of every single image at every position and also at the GaP/AlP bottom interface. The larger lattice constant within the QD (compared to the lattice constant of uncleaved QD with a composition of In_0.3_Ga_0.7_As_0.85_Sb_0.15_ which is 0.58404 nm) is the result of high compressive strain, which distorts the cleaved surface. The effect of compressive strain can be clearly seen in Fig. 7a, as a sharp drop in the lattice constant above and below the QD. As mentioned before, most of the GaAs is consumed during the QD formation, and we suppose there might be about a ML of GaAs present at the bottom of the QDs acting as a sort of wetting layer. FE simulations were performed using the composition estimate discussed above. In the simulation for a QD with an average composition of In_0.3_Ga_0.7_As_0.85_Sb_0.15_, we observed a reasonable fit to the experimental lattice constant profile as shown in Fig. 7a. Complete details on FE simulation are given in the section S-1 of the Supplemental Material. The effect of varying In concentration on the fitting of the local lattice constant is given in Fig. S4 in section S-1 of the Supplemental Material.

The outward relaxation of the cleaved QD strongly depends on size, composition, and overall strain distribution, which can be easily measured by X-STM height profiles shown in Fig. 7b. Efforts have been made to fit the outward relaxation of the cleaved surface with the one calculated from FE simulation; however, the results are unsuccessful, the reported composition of ∼In_0.3_Ga_0.7_As_0.85_Sb_0.15_ gives a relaxation difference of 80–100 pm, see Fig. 7b. The exact reason for this difference in relaxation is not known yet but, we suppose that the discrepancy can be partially explained by the following reasons: (1) due to the high density of the QDs, most of the QDs share 1–2 BLs at the bottom (see Fig. 1), which can affect the strain distribution and thus the outward relaxation of the cleaved surface; (2) multiple layers with different lattice constants could act differently for the simulation creating more room for error; (3) since GaP is an indirect semiconductor, the electronic effects could influence the height profile measured by the STM tip. High negative bias voltages have been used to suppress the electronic contribution but still, that did not help to resolve the issue (see section S-2 of Supplemental Material for the effect of bias voltage on outward relaxation of the QD). However, the tunnel conditions of the X-STM might be altered when the tip scans from the indirect bandgap material of the cladding (GaP) to the direct bandgap material of the QD, i.e., (InGa)(AsSb).

### QDs: capping layer

**Fig. 8:**
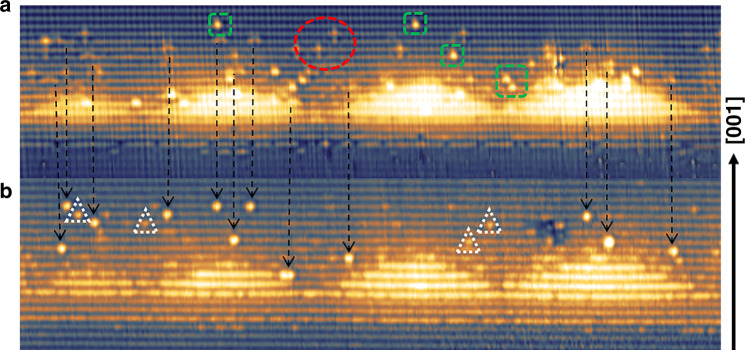
40 × 11 nm2 X-STM images of the same area. **a** Empty-state image taken at Vb = +3.0 V, It = 90 pA. **b** Filled-state image taken at Vb = −3.65 V, It = 90 pA. The black arrows indicate the position of Sb atoms in both images, the white triangles indicate some of the subsurface Sb atoms in the filled-state image, the green squares indicate some of the segregated surface In atoms, and the red circle indicates some subsurface In atoms. The dark to bright contrast difference is 250 pm and the arrow on the right side indicates the growth direction [001]

Fig. 8 shows both filled and empty-state images of the same area taken at *V*_b_ = −3.65 V and *V*_b_ = +3.0 V revealing group V (P, As, Sb) and group III (In, Al, Ga) sublattice, respectively. Tilley et al.^83^ reported simulated filled and empty-state images of isovalent impurities (including Sb) in GaAs. We observed similar features for both segregated Sb and In in our experiment, which further supports our results.

In Fig. 8, in empty-state imaging, the Sb atoms are identified as two-lobed features whereas, in filled-state imaging, the same atoms look like large bright spots (indicated by black arrows). The white dashed triangles in the filled-state image indicate a few subsurface Sb atoms that give rise to a weaker contrast^75^. In the empty-state image, the green squares represent surface indium atoms and within the red circle, the star-like features can be seen that are not visible in the filled-state image, which we identify as subsurface In atoms.

**Fig. 9:**
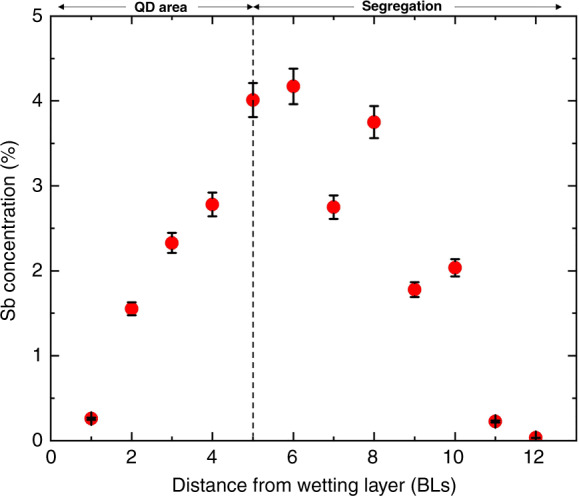
Sb concentration as a function of distance in BLs from the wetting layer in the growth direction [001]. The distance from 0 to the dotted line along the *x*-axis represents the area occupied by the QDs, the dotted line being the top of the QDs. The Sb atoms are counted over a distance of 1 μm on each atomic row, using multiple X-STM filled-state images

It is clear that the bright atomic features in the 6 nm capping layer of the filled-state images are individual Sb atoms segregated from the QD layer. This is supported by the fact that Sb is the only anionic element deposited during the QD growth and also, as mentioned in the APT results, the Sb peak lies outside the QD region indicating the presence of Sb above the QDs, see Figs. 4 and 5a. A full distribution of segregated Sb atoms is shown in Fig. 9, where the Sb atoms are counted over a lateral distance of 1 µm using multiple X-STM filled-state images. These Sb atoms were not incorporated during the QD growth and segregated into the capping layer. The mechanism of Sb segregation into the capping layer is well known, and it has been also observed for GaSb/GaP QDs grown by MBE^84^. A similar concentration of In atoms is identified in the capping layer, segregated from QDs. Due to the limited number of empty-state images obtained during the experiment, the In segregation profile is not shown here but, looking at Fig. 8, one can easily identify a similar number of In and Sb in the capping layer.

The area from the wetting layer to the 5^th^ BL (from 0 to the dotted line along the *x*-axis) is mostly occupied by QDs, and few Sb atoms are found in between QDs creating a linear increase in the concentration with the distance. One can observe almost an exponential decay trend (typical signature of the segregation) in Sb concentration after the sixth BL. The Sb atoms segregated utmost to 11 BLs (∼6.0 nm) from the wetting layer, which corresponds exactly to the position where the temperature was raised during growth to remove the segregated atoms from the surface and to grow an additional capping layer. Therefore, from Figs. 8 and 9, a maximum concentration of ∼3% was found for segregated Sb and In integrated over the whole capping layer (∼6 nm from the wetting layer).

## Discussion

The structural and compositional characterization of these QDs is important as the QD-Flash storage time strongly depends on localization potential and capture cross-section^25^, which in turn depends on the size, shape, and composition of the QDs. Larger QD dimensions increase the localization depth at the expense of capture cross-section. So, there is a critical interdependence between a large enough localization potential and a small enough capture cross-section to obtain the desired device properties.

In “QDs: size and shape“ section, we estimated the size and shape of the QDs from many filled-state topographic images and Bruls model^76,77^ based on few assumptions, such as QDs with a flattop facet, uniform size, and shape of the QDs, which are slightly different from reality. As already mentioned in “QDs: size and shape“ section, few QDs are identified with a trapezoidal shape (cleaved through the QD center) with a very small top facet, while most of the QDs appeared to be near triangles (cleaved far from the QD center) in the X-STM images. Among the 261 QD images, only 7–8% of the QDs are identified with a 2.0–3.0 nm flattop facet (trapezoidal shape) and the rest are near triangular shaped. Ideally, depending on cleaving position, the height vs base length shown in Fig. 2 should fall on a single line (for example red line in Fig. 2), the broad distribution of height vs base lengths suggests an inhomogeneity in QDs size and shape. The deviation of the measured height from the linear fit (Δ*H*) is ~±0.5 nm (detailed explanation is provided in section S-4 of the Supplemental Material). We suppose that this small error could arise either from the inaccuracy in X-STM measurement due to thermal drift of the X-STM tip or from the size and shape inhomogeneities of the QDs. Since the surfactant effect of Sb reduces the adatom mobility on the growth surface leading to smaller and uniform QDs^27,71^, we assumed that the QDs are square-based pyramids with very little inhomogeneities in QDs size and shape.

The Bruls model^76^ is used to determine the orientation of QDs with respect to the cleaving plane, whether it is parallel to the base or diagonal of the QD. The strain tensor of the growth surface is always enforcing, that the edges of all QDs are aligned in <0 0 1> directions of the crystal plane, defining a minimum of built-in strain energy^85,86^. Thus entropy or kinetics can cause small variations of the shape or volume between individual dots, but not their alignment with respect to the cleaving plane. The AFM analysis of uncapped QDs reported a height of ~4.0 nm and a similar height of ~3.5 nm is observed from transmission electron microscopy (TEM)^26,27^. A maximum height of 3.0–3.5 nm was found by X-STM, the difference in height of the QDs measured by AFM and X-STM can be attributed to the dissolution of QD apex due to the strain-induced intermixing of QD material with the capping layer. QDs with base lengths in the order of ~30–50 nm were reported from the AFM analysis. However, the measurement of base lengths from AFM is cumbersome as it strongly suffers from the AFM tip convolution^87,88^. On the other hand, the base length reported by TEM (~15 nm) is in good agreement with our X-STM analysis.

It is difficult to identify the exact Miller indices of QD side facets but, for a QD in Fig. 3, we can measure the angle between the side facets and the base plane (001) and estimate the Miller indices of side facets. The angle for both side facets is close to 38 ± 2° with respect to the base plane (001). The closest crystallographic plane which makes an angle of ∼35° with (001) is {112}. It has been reported that the InAs/GaAs and InAs/InP QDs grown on low-index substrates, such as (001), tends to have high-index side facets, such as {136} and {137}, corresponding with an angle of ~28° (refs. ^89,90^). The exact indices of the QD side facets can also depend on surface reconstruction and growth kinetics. With respect to our dots, it is useful to note that {136} facets were also observed for InGaAs QDs grown on GaP^91^. We can only provide a crude approximation and thus do not want to make a conclusive remark on the exact indices of the side facets.

APT analysis suggested a 2:1 ratio of In:Sb and a decent fit to the lattice constant profile from FE simulations was obtained with a composition of 30% In and 15% Sb, but a lower Sb incorporation is possible due to its surfactant effect. It is clearly visible in Fig. 5a, where the Sb peak started after In and most of the Sb peak lies outside the QD region. Optical studies were performed on similar samples by Steindl et al.^40,67^ by using excitation and temperature-dependent PL in relation to the electronic structure, in combination with ***k.p*** calculations. Detailed theoretical analysis of the electronic structure of these (InGa)(AsSb)/GaAs/GaP QDs obtained using ***k.p*** calculations, comparing the QDs on both GaP and GaAs substrates are reported in ref. ^32^. The hole localization energy calculated was further supported by the deep-level transient spectroscopy performed on the same sample used for the current analysis^27^. Both studies reported a composition of ∼In_x_Ga_1 − *x*_As_1 − *y*_Sb_*y*_, where *x* = 0.20 and y = 0.10–0.20, which is very close to the composition analysis reported in this work. We observed a very good agreement between all the experimental (X-STM, APT, optical, and electrical measurements) and theoretical studies, regarding the QDs composition, providing a full description of this novel and complex QD system.

In summary, we analyzed the size, shape, and composition of the highly strained (InGa)(AsSb) SKQDs embedded in a GaP matrix with atomic resolution by X-STM and APT. We found that these QDs have a truncated pyramid shape with a height of 3.0 ± 0.5 nm and a base length of 8.5 ± 0.6 nm. Most of the QDs are close to near pyramids with a small top facet indicating that the apex of the pyramids might have been dissolved during the capping. Detailed composition analysis was performed by using APT. FE simulations were carried out by using structural data from X-STM, and reasonably good fits were obtained for the local lattice constant profile of the cleaved QDs. FE simulations together with X-STM results are used to estimate the composition of the QDs, which is close to ∼In_x_Ga_1 − *x*_As_1 − *y*_Sb_*y*_, where *x* = 0.25–0.30 and *y* = 0.10–0.15. A ratio of 2:1—In:Sb was observed from atom probe experiments, which is in good agreement with X-STM, FE simulations, optical, electrical, and also the theoretical studies^27,32,40,67^. These results prove that the InGaSb and GaAs layers have strongly intermixed during the dot formation process. Finally, we reported a detailed analysis of the capping layer and observed maximum ∼3% of Sb and In segregated from the QD layer into the capping layer. With the present study, we are able to confirm the previous results on optical, electrical, and theoretical studies, suggesting a limited Sb incorporation into QDs supporting the surfactant behavior of Sb during the dot formation process. Overall, the current study provides a detailed structural and compositional overview of these novel QDs, shedding light on the Sb incorporation into the QD layer and provides valuable feedback to the MOVPE growth of Sb-based QDs, to optimize the storage time of QD-Flash memory devices or the QDs optical activity^67^.

## Materials and methods

### Sample growth menu

**Fig. 10:**
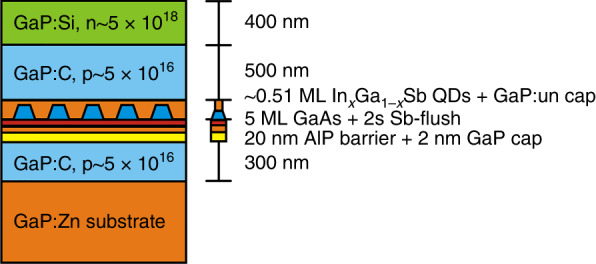
Schematic structure of the X-STM sample grown by MOVPE

The sample was grown in a horizontal Aixtron 200 MOVPE reactor on a *p*-doped GaP(001) substrate using H_2_ as a carrier gas, as also outlined in ref. ^27^. Growth commenced with a 300 nm thick GaP:C (doping concentration *n* = 5 × 10^16^ cm^−3^) buffer layer grown at 620 °C. The substrate temperature was increased to 800 °C in order to grow a 20 nm thick AlP barrier layer, which increases the hole localization energy of QDs^38^, followed by a 2 nm GaP cap. Subsequently, the temperature was reduced to 500 °C and kept constant to grow 5 MLs of GaAs IL, which facilitates the SK growth of QDs. A 2 s Sb-flush was applied prior to the deposition of QDs, with an input flux of 2.5 µmol min^−1^. The Sb acts as a surfactant and can affect the QD formation, leading to smaller and more homogeneous (InGa)(AsSb) QDs^25^. A 0.51 ML InGaSb corresponding to a growth rate of 0.15 ML s^−1^ was supplied for the formation of QDs. After a GRI of 1 s, a thin cap of GaP (6 nm) was grown at 500 °C, thereafter the temperature was raised to 620 °C to grow an additional 16 nm of undoped GaP. Finally, a 500 nm of GaP:C (*n* = 5 × 10^16^ cm^−3^) and an additional 400 nm of GaP:Si (*n* = 5 × 10^18^ cm^−3^) were grown at 620 °C to finish the *p*–*n* diode structure. A schematic structure of the sample used for X-STM measurement is shown in Fig. 10. We note that the sample, investigated here with X-STM, is exactly the same as the one with a storage time record of 1 h at room temperature, measured via deep-level transient spectroscopy^27^.

### Cross-sectional scanning tunneling microscopy

All the X-STM measurements were performed in a conventional Omicron low-temperature STM at liquid nitrogen temperature (77 K) under ultrahigh vacuum (UHV; 4–6 × 10^−11^ mbar). The measurements were carried out on a clean {110} surface freshly obtained by cleaving the sample in UHV. STM tips were made of polycrystalline tungsten wires obtained by electrochemical etching followed by baking and Ar sputtering inside the STM preparation chamber in UHV. All the filled and empty-state images of the QDs were acquired in constant current mode. For filled-state imaging at high negative bias voltages, P, As, and Sb sublattices (group V) were imaged, while in empty-state imaging at positive bias voltages, In, Ga, and Al sublattices (group III) were imaged. Due to the atomic arrangement of the {110} surfaces of zincblende crystals, only every second ML along the growth direction is visible in the X-STM images^92^.

### Atom probe tomography

Standard atom probe sample lift-out and sharpening was carried out using a dual-beam Focused Ion Beam/Scanning Electron Microscope (Zeiss Crossbeam 400 (ref. ^93^)). QD containing multilayer structure was observed ~900–1000 nm below the surface as a line of low contrast. Samples were sharpened using 30 kV Ga^+^ annular milling and energy-dispersive electron spectroscopy^94^ was carried out on the partially sharpened tip, confirming the presence of aluminum below the low contrast line. A 2 kV Ga^+^ polishing was then used to place the apex of the sample ~100 nm above this line. The samples were analyzed using a Cameca LEAP5000 XS atom probe system with a laser wavelength of 355 nm and a detection efficiency of 80%. The analysis was carried out using a 200 kHz pulse repetition rate, 5 fJ pulse energy, 55 K base temperature, a base pressure <3 × 10^−11^ torr, and a detection rate of 1% (where the detection rate is the percentage of laser pulses per second that resulted in a detectable ion impact). Reconstruction of the obtained data was carried out using IVAS 3.8.2 software and using a shank angle approach, optimized for the upper AlP layer to be flat and to match the known thickness as measured by X-STM.

## Supplementary information

Supplemental Material

## References

[1] Bimberg D (1997). InGaAs-GaAs quantum-dot lasers. IEEE J. Sel. Top. Quant. Electron..

[2] Bimberg D (2005). Quantum dots for lasers, amplifiers and computing. J. Phys. D Appl. Phys..

[3] Müller T (2018). A quantum light-emitting diode for the standard telecom window around 1,550 nm. Nat. Commun..

[4] Martín-Sánchez J (2009). single photon emission from site-controlled InAs quantum dots grown on GaAs(001) patterned substrates. ACS Nano.

[5] Yuan ZL (2002). Electrically driven single-photon source. Science.

[6] Salter CL (2010). An entangled-light-emitting diode. Nature.

[7] Aberl J (2017). Inversion of the exciton built-in dipole moment in In(Ga)As quantum dots via nonlinear piezoelectric effect. Phys. Rev. B.

[8] Huber D (2019). Single-particle-picture breakdown in laterally weakly confining GaAs quantum dots. Phys. Rev. B.

[9] Csontosová D, Klenovský P (2020). Theory of magneto-optical properties of neutral and charged excitons in GaAs/AlGaAs quantum dots. Phys. Rev. B.

[10] Klenovský P (2015). Polarization anisotropy of the emission from type-II quantum dots. Phys. Rev. B.

[11] Schimpf C (2019). Resolving the temporal evolution of line broadening in single quantum emitters. Opt. Express.

[12] Steindl P (2021). Artificial coherent states of light by multiphoton interference in a single-photon stream. Phys. Rev. Lett..

[13] Nozik AJ (2002). Quantum dot solar cells. Phys. E Low. Dimens. Syst. Nanostruct..

[14] Michler, P. *Quantum Dots for Quantum Information Technologies* (Springer, 2017).

[15] Hadfield RH (2009). Single-photon detectors for optical quantum information applications. Nat. Photonics.

[16] Li XQ (2003). An all-optical quantum gate in a semiconductor quantum dot. Science.

[17] Křápek V (2010). Quantum entanglement in lateral GaAs/AlGaAs quantum dot molecules. J. Phys. Conf. Ser..

[18] Klenovský P, Křápek V, Humlíček J (2016). Type-II InAs/GaAsSb/GaAs quantum dots as artificial quantum dot molecules. Acta Phys. Pol. A.

[19] Klenovský P (2018). Effect of second-order piezoelectricity on the excitonic structure of stress-tuned In(Ga)As/GaAs quantum dots. Phys. Rev. B.

[20] Alonso-Álvarez D (2007). Optical investigation of type II GaSb/GaAs self-assembled quantum dots. Appl. Phys. Lett..

[21] Klenovský P (2010). Modelling of electronic states in InAs/GaAs quantum dots with GaAsSb strain reducing overlayer. J. Phys. Conf. Ser..

[22] Klenovský P (2010). Electronic structure of InAs quantum dots with GaAsSb strain reducing layer: Localization of holes and its effect on the optical properties. Appl. Phys. Lett..

[23] Klenovský P, Steindl P, Geffroy D (2017). Excitonic structure and pumping power dependent emission blue-shift of type-II quantum dots. Sci. Rep..

[24] Rautert J (2019). Optical orientation and alignment of excitons in direct and indirect band gap (In,Al)As/AlAs quantum dots with type-I band alignment. Phys. Rev. B.

[25] Bimberg, D., Mikolajick, T. & Wallart, X. Novel quantum dot based memories with many days of storage time: last steps towards the holy grail? In *Proceedings of the19th Non-Volatile Memory Technology Symposium (NVMTS)*, 1–4 (IEEE, 2019).

[26] Sala, E. M. et al. Growth and structure of In_0.5_Ga_0.5_Sb quantum dots on GaP(001). *Appl. Phys. Lett.* **109**, 102102 (2016).

[27] Sala EM (2018). MOVPE-growth of InGaSb/AlP/GaP(001) quantum dots for nanoscale memory applications. Phys. Status Solidi.

[28] Bimberg, D. et al. Antimony-based quantum dot memories. In *Proceedings of SPIE 7947, Quantum Dots and Nanostructures: Synthesis, Characterization, and Modeling VIII*, 79470L (SPIE, 2011).

[29] Marent A, Geller M, Bimberg D (2009). A novel nonvolatile memory based on self-organized quantum dots. Microelectron. J..

[30] Geller M (2008). A write time of 6ns for quantum dot–based memory structures. Appl. Phys. Lett..

[31] Geller, M., Marent, A.&Bimberg, D. in *6 Handbook of Nanophysics* (ed. Sattler, K. D.) (CRC, 2010).

[32] Klenovský P, Schliwa A, Bimberg D (2019). Electronic states of (InGa)(AsSb)/GaAs/GaP quantum dots. Phys. Rev. B.

[33] Nowozin, T. *Self-Organized Quantum Dots for Memories* (Springer, 2014).

[34] Grassman TJ (2013). Nucleation-related defect-free GaP/Si(100) heteroepitaxy via metal-organic chemical vapor deposition. Appl. Phys. Lett..

[35] Volz K (2011). GaP-nucleation on exact Si (001) substrates for III/V device integration. J. Cryst. Growth.

[36] Marent, A. et al. 10^6^ years extrapolated hole storage time in GaSb∕AlAs quantum dots. *Appl. Phys. Lett.* **91**, 242109 (2007).

[37] Bonato L (2016). Hole localization energy of 1.18 eV in GaSb quantum dots embedded in GaP. Phys. Status Solidi.

[38] Bonato, L. et al. 230 s room-temperature storage time and 1.14 eV hole localization energy in In_0.5_Ga_0.5_As quantum dots on a GaAs interlayer in GaP with an AlP barrier. *Appl. Phys. Lett.* **106**, 042102 (2015).

[39] Sala, E. M. *Growth and Characterization of Antimony-based Quantum Dots in GaP Matrix for Nanomemories*. PhD thesis, Technische Universität Berlin, 2018.

[40] Steindl P (2019). Optical response of (InGa)(AsSb)/GaAs quantum dots embedded in a GaP matrix. Phys. Rev. B.

[41] Gong Q (2004). Capping process of InAs/GaAs quantum dots studied by cross-sectional scanning tunneling microscopy. Appl. Phys. Lett..

[42] Ulloa, J. M., Offermans, P. & Koenraad, P. M. in *Handbook of Self Assembled Semiconductor Nanostructures for Novel Devices in Photonics and Electronics* (ed. Henini, M.) (Elsevier, 2008).

[43] Keizer JG (2010). Atomic scale analysis of self assembled GaAs/AlGaAs quantum dots grown by droplet epitaxy. Appl. Phys. Lett..

[44] Bruls DM (2003). Stacked low-growth-rate InAs quantum dots studied at the atomic level by cross-sectional scanning tunneling microscopy. Appl. Phys. Lett..

[45] Keizer JG (2011). Structural atomic-scale analysis of GaAs/AlGaAs quantum wires and quantum dots grown by droplet epitaxy on a (311)A substrate. Appl. Phys. Lett..

[46] Gajjela RSR (2020). Cross-sectional scanning tunneling microscopy of InAs/GaAs(001) submonolayer quantum dots. Phys. Rev. Mater..

[47] Gajjela RSR, Koenraad PM (2021). Atomic-scale characterization of droplet epitaxy quantum dots. Nanomaterials.

[48] Bocquel J (2014). Composition profiling of GaAs/AlGaAs quantum dots grown by droplet epitaxy. Appl. Phys. Lett..

[49] Keizer JG (2011). Shape control of quantum dots studied by cross-sectional scanning tunneling microscopy. J. Appl. Phys..

[50] Offermans P (2005). Formation of InAs quantum dots and wetting layers in GaAs and AlAs analyzed by cross-sectional scanning tunneling microscopy. Phys. E Low. Dimens. Syst. Nanostruct..

[51] Keizer JG (2012). Atomically resolved study of the morphology change of InAs/GaAs quantum dot layers induced by rapid thermal annealing. Appl. Phys. Lett..

[52] Blokland JH (2009). Ellipsoidal InAs quantum dots observed by cross-sectional scanning tunneling microscopy. Appl. Phys. Lett..

[53] Offermans P (2005). Atomic-scale structure and photoluminescence of InAs quantum dots in GaAs and AlAs. Phys. Rev. B.

[54] He J (2004). Formation of columnar (In,Ga)As quantum dots on GaAs(100). Appl. Phys. Lett..

[55] Offermans P (2003). Annealing of InGaAlAs digital alloy studied with scanning-tunneling microscopy and filled-states topography. Appl. Phys. Lett..

[56] Fain B (2010). Electronic structure of cleaved InAsP/InP(001) quantum dots measured by scanning tunneling spectroscopy. Appl. Phys. Lett..

[57] Gaan S (2010). Electronic states of InAs/GaAs quantum dots by scanning tunneling spectroscopy. Appl. Phys. Lett..

[58] Gaan S (2010). Size, shape, composition, and electronic properties of InAs/GaAs quantum dots by scanning tunneling microscopy and spectroscopy. J. Appl. Phys..

[59] Plantenga RC (2017). Spatially resolved electronic structure of an isovalent nitrogen center in GaAs. Phys. Rev. B.

[60] Krammel CM (2017). Incorporation of Bi atoms in InP studied at the atomic scale by cross-sectional scanning tunneling microscopy. Phys. Rev. Mater..

[61] Krammel CM (2018). Structural and electronic properties of isovalent boron atoms in GaAs. J. Appl. Phys..

[62] Krammel CM (2020). Probing the local electronic structure of isovalent Bi atoms in InP. Phys. Rev. B.

[63] Koenraad PM, Flatté ME (2011). Single dopants in semiconductors. Nat. Mater..

[64] Tjeertes D (2020). N − *n*H complexes in GaAs studied at the atomic scale by cross-sectional scanning tunneling microscopy. Phys. Rev. B.

[65] Giddings AD (2011). Composition profiling of InAs quantum dots and wetting layers by atom probe tomography and cross-sectional scanning tunneling microscopy. Phys. Rev. B.

[66] Müller M (2008). Atomic scale characterization of buried In_*x*_Ga_1−*x*_As quantum dots using pulsed laser atom probe tomography. Appl. Phys. Lett..

[67] Steindl, P. et al. On the importance of antimony for temporal evolution of emission from self-assembled (InGa)(AsSb)/GaAs quantum dots on GaP(001). Preprint at https://arxiv.org/abs/2101.06299 (2021).

[68] Ebert P (1995). Formation of anion vacancies by Langmuir evaporation from InP and GaAs (110) surfaces at low temperatures. Phys. Rev. B.

[69] Prohl, C. et al. Spatial structure of In_0.25_Ga_0.75_As/GaAs/GaP quantum dots on the atomic scale. *Appl. Phys. Lett.* **102**, 123102 (2013).

[70] Guimard D (2009). Interface properties of InAs quantum dots produced by antimony surfactant-mediated growth: Etching of segregated antimony and its impact on the photoluminescence and lasing characteristics. Appl. Phys. Lett..

[71] Guimard D (2007). Effect of antimony on the density of InAs/Sb:GaAs(100) quantum dots grown by metalorganic chemical-vapor deposition. J. Cryst. Growth.

[72] Gong Q (2002). Leveling and rebuilding: An approach to improve the uniformity of (In,Ga)As quantum dots. Appl. Phys. Lett..

[73] Lian GD (1998). Modification of InAs quantum dot structure by the growth of the capping layer. Appl. Phys. Lett..

[74] Costantini G (2006). Interplay between thermodynamics and kinetics in the capping of InAs/GaAs(001) quantum dots. Phys. Rev. Lett..

[75] Ebert HP (2003). Imaging defects and dopants. Mater. Today.

[76] Bruls DM (2002). Determination of the shape and indium distribution of low-growth-rate InAs quantum dots by cross-sectional scanning tunneling microscopy. Appl. Phys. Lett..

[77] Bruls DM (2001). Cracking self-assembled InAs quantum dots. Appl. Phys. A.

[78] Hellman OC (2000). Analysis of three-dimensional atom-probe data by the proximity histogram. Microsc. Microanal..

[79] Larson, D. J. et al. *Local Electrode Atom Probe Tomography* (Springer, 2013).

[80] London AJ (2019). Quantifying uncertainty from mass-peak overlaps in atom probe microscopy. Microsc. Microanal..

[81] Marquis EA (2011). Evolution of tip shape during field evaporation of complex multilayer structures. J. Microsc..

[82] Koelling S (2009). High depth resolution analysis of Si/SiGe multilayers with the atom probe. Appl. Phys. Lett..

[83] Tilley FJ (2016). Scanning tunneling microscopy contrast of isovalent impurities on the GaAs (110) surface explained with a geometrical model. Phys. Rev. B.

[84] Desplanque L (2017). Morphology and valence band offset of GaSb quantum dots grown on GaP(001) and their evolution upon capping. Nanotechnology.

[85] Heinrichsdorff F (2000). High-power quantum-dot lasers at 1100 nm. Appl. Phys. Lett..

[86] Shchukin, V. A., Ledentsov, N. N. & Bimberg, D. *Epitaxy of Nanostructures* (Springer, 2004)

[87] Tranchida D, Piccarolo S, Deblieck RAC (2006). Some experimental issues of AFM tip blind estimation: the effect of noise and resolution. Meas. Sci. Technol..

[88] Canet-Ferrer J (2014). Correction of the tip convolution effects in the imaging of nanostructures studied through scanning force microscopy. Nanotechnology.

[89] Jacobi K (2003). Atomic structure of InAs quantum dots on GaAs. Prog. Surf. Sci..

[90] Yoon S (1999). Shape change of InAs self-assembled quantum dots induced by As/P exchange reaction. Thin Solid Films.

[91] Robert C (2012). Electronic, optical, and structural properties of (In,Ga)As/GaP quantum dots. Phys. Rev. B.

[92] Feenstra RM (1987). Atom-selective imaging of the GaAs(110) surface. Phys. Rev. Lett..

[93] Thompson K (2007). In situ site-specific specimen preparation for atom probe tomography. Ultramicroscopy.

[94] Gopon P (2019). Complementary SEM-EDS/FIB-SEM sample preparation techniques for atom probe tomography of nanophase-Fe 0 in Apollo 16 regolith sample 61501,22. Microsc. Microanal..

