# Supplementary material for: Structural and compositional analysis of (InGa)(AsSb)/GaAs/GaP Stranski–Krastanov quantum dots
Source: Light Sci Appl. 2021 Jun 15;10:125. doi: 10.1038/s41377-021-00564-z (PMC8203795; doi:10.1038/s41377-021-00564-z)
Supplement: Supplementary file 1 — Supplemental Material [file 41377_2021_564_MOESM1_ESM.pdf]

Structural and Compositional Analysis of (InGa)(AsSb)/GaAs/GaP Stranski-Krastanov Quantum Dots

Raja S. R. Gajjela[1,*], Arthur L. Hendriks[1], James O. Douglas[2], Elisa M. Sala[3,4], Petr Steindl[5,6], Petr Klenovsky`[5,7], Paul A. J. Bagot[2], Michael P. Moody[2], Dieter Bimberg[8,3], and Paul M. Koenraad[1]

[1] Department of Applied Physics, Eindhoven University of Technology, Eindhoven 5612 AZ, The Netherlands

[2]Department of Materials, University of Oxford, Parks Road, Oxford, OX1 3PH, UK

[3] Center for Nanophotonics, Institute for Solid State Physics, Technische Universität Berlin, Hardenbergstr. 36, 10623 Berlin, Germany

[4] EPSRC National Epitaxy Facility, The University of Sheffield, North Campus, Broad Lane, S3 7HQ Sheffield, United Kingdom

[5] Department of Condensed Matter Physics, Faculty of Science, Masaryk University, Kotlářská 267/2, 61137 Brno, Czech Republic

[6] Huygens-Kamerlingh Onnes Laboratory, Leiden University, P.O. Box 9504, 2300 RA Leiden, Netherlands

[7] Czech Metrology Institute, Okružní 31, 63800 Brno, Czech Republic

[8] "Bimberg Chinese-German Center for Green Photonics" Changchun Institute of Optics, Fine Mechanics and Physics, Chinese Academy of Sciences at CIOMP, 13033 Changchun, China

*r.s.r.gajjela@tue.nl

S-1: Finite Element Simulation

Simulations were performed with the finite element method (FEM) using the program: COMSOL Multiphysics to model the strain profile and surface relaxation of the quantum dots (QDs). COMSOL is a useful tool to simulate the outward relaxation and the local lattice constant using the solid-mechanics module. This can be applied in the determination of the material composition within a quantum well (QW) or quantum dot (QD) by comparing experimental and simulated results.

COMSOL numerically solves differential equations based on continuum elasticity theory. To start the simulation, the program needs a certain geometry of the QD or QW with the appropriate initial strain matrix. The initial strain will deform the cubic volume elements by acting as a force on their surfaces from different directions. The initial strain is caused by the lattice mismatch between the substrate material and the epitaxially grown layers. This lattice mismatch is given by:

$$\epsilon_0 = \frac{a - a_0}{a_0} \qquad \textit{Equation 1}$$

where a_0 is the original lattice constant of the substrate and a is the lattice constant of the epitaxially grown layers.

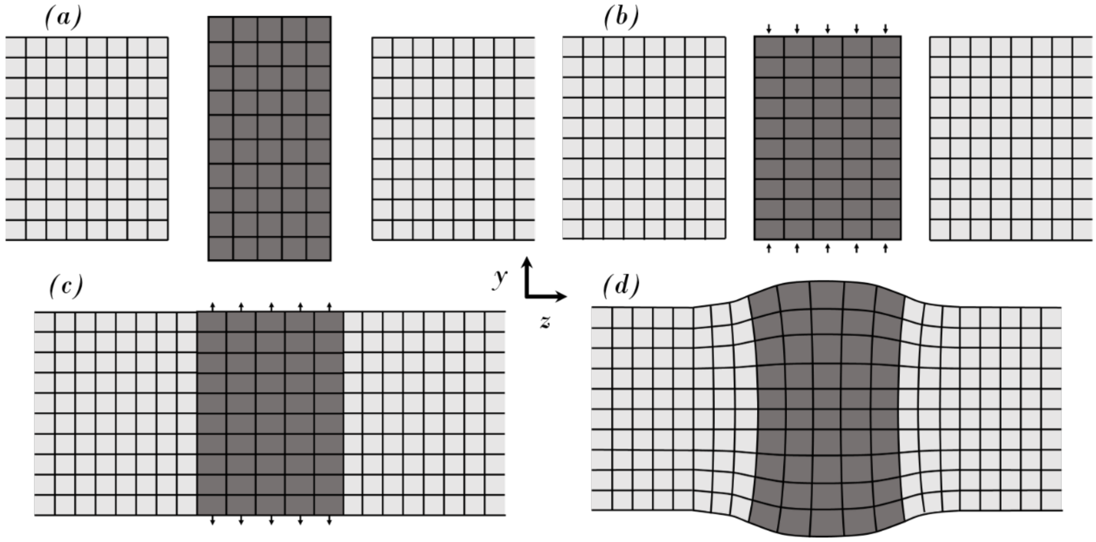

Figure S1: Schematic model of a strained quantum well at a surface. The QW has a higher lattice constant which is strained to be equal to the cladding. When cleaved (d) the QW relaxes outwards. Modified from [61].

In Figure S1, the four-step approach for a strained QW is shown: (a) QW and two cladding layers are brought close to each other. The QW's lattice constant exceeds the lattice constant of the cladding layers by the fraction ϵ_0 as defined by Equation 1. Here z is the growth direction, and y is the outward normal to the cleaved surface; (b) The lattice constant of the QW is now matched to the lattice constant of the cladding. Uniform stress in the x and y-direction decreases the lattice constant by ϵ_0. Due to the reduction of lattice constant in x and y, the lattice constant in the z-direction will increase as defined by the Poisson ratio $'\nu'$; (c) Now the QW is joined with the cladding layers without any additional strain. The initial strain of a QW is defined to be:

$$\epsilon_{QW} = \begin{bmatrix} \epsilon_{xx} & \epsilon_{xy} & \epsilon_{xz} \\ \epsilon_{yx} & \epsilon_{yy} & \epsilon_{yz} \\ \epsilon_{zx} & \epsilon_{zy} & \epsilon_{zz} \end{bmatrix} = \begin{bmatrix} -\epsilon_0 & 0 & 0 \\ 0 & -\epsilon_0 & 0 \\ 0 & 0 & \frac{2\nu\epsilon_0}{1-\nu} \end{bmatrix}$$ *Equation 2*

where ϵ_0 is the lattice mismatch, ν is the Poisson's ratio (which is a material property), and z is the growth direction[1,2]; (d) when the material is cleaved, outward pressure is applied to the exposed surface. This will displace the surface of both the cladding layers and the QW, which is the relaxation that can be experimentally measured with an X-STM.

For a QD, the approach is slightly different, as seen in Figure S2. This due to the change in the shape of the structure. Instead of a full slab, only a small volume with limited dimensions has to be fitted inside the cladding material. Therefore, the lattice constant of the full QD is reduced equally in all three dimensions (x,y,z) by the lattice mismatch to match the lattice constant of the cladding. In matrix form this is written as:

$$\epsilon_{QW} = \begin{bmatrix} \epsilon_{xx} & \epsilon_{xy} & \epsilon_{xz} \\ \epsilon_{yx} & \epsilon_{yy} & \epsilon_{yz} \\ \epsilon_{zx} & \epsilon_{zy} & \epsilon_{zz} \end{bmatrix} = \begin{bmatrix} -\epsilon_0 & 0 & 0 \\ 0 & -\epsilon_0 & 0 \\ 0 & 0 & -\epsilon_0 \end{bmatrix}$$ *Equation 3*

The elastic properties are described with 3 independent values (C_{11}, C_{12}, and C_{44}) in a simple cubic-symmetric case given by the stiffness matrix (D):

$$D = \begin{bmatrix} C_{11} & C_{12} & C_{12} & 0 & 0 & 0 \\ C_{12} & C_{11} & C_{12} & 0 & 0 & 0 \\ C_{11} & C_{12} & C_{12} & 0 & 0 & 0 \\ 0 & 0 & 0 & C_{44} & 0 & 0 \\ 0 & 0 & 0 & 0 & C_{44} & 0 \\ 0 & 0 & 0 & 0 & 0 & C_{44} \end{bmatrix}$$ *Equation 4*

This elasticity matrix is then used in the stress-strain relation as:

$$\sigma = D\,\epsilon$$ *Equation 5*

where the stress and strain are 6×1 vectors:

$$\sigma = \begin{bmatrix} \sigma_{xx} \\ \sigma_{yy} \\ \sigma_{zz} \\ \sigma_{xy} \\ \sigma_{yz} \\ \sigma_{zx} \end{bmatrix}, \epsilon = \begin{bmatrix} \epsilon_{xx} \\ \epsilon_{yy} \\ \epsilon_{zz} \\ \epsilon_{xy} \\ \epsilon_{yz} \\ \epsilon_{zx} \end{bmatrix}$$ *Equation 6*

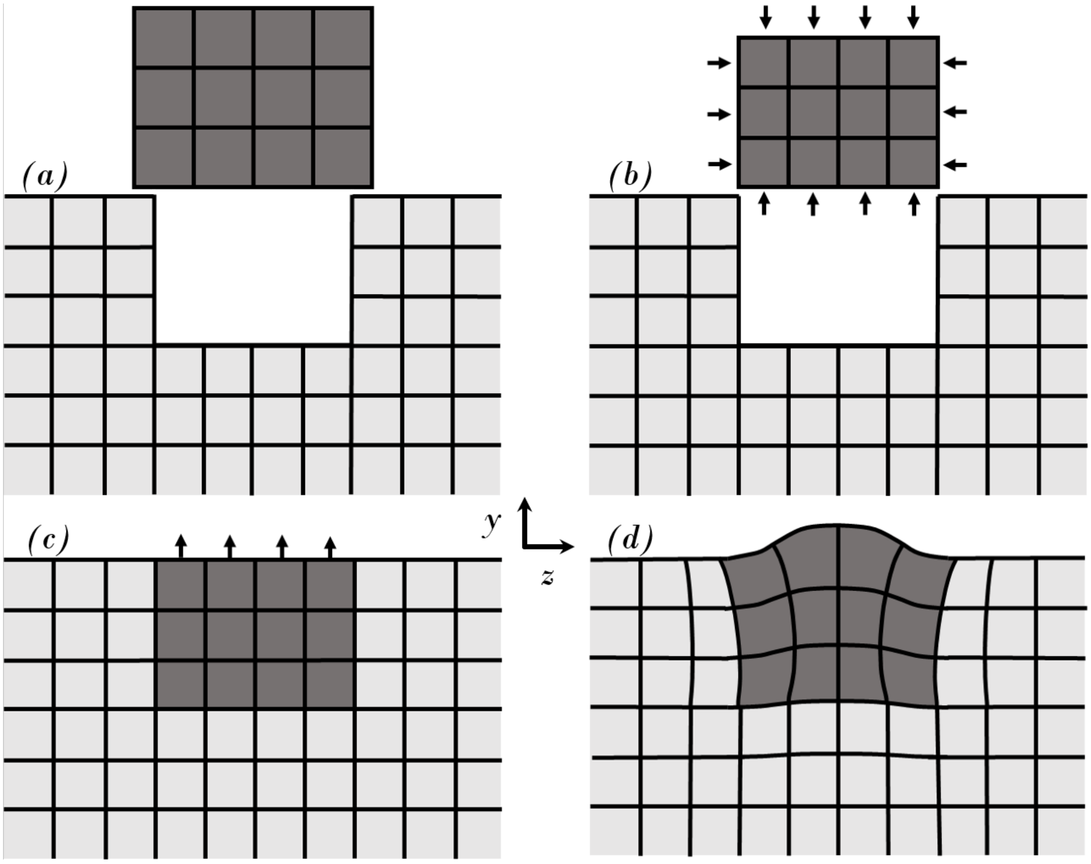

Figure S2: Schematic model of a strained quantum dot at a surface. Here, the higher lattice constant of the QD is strained equally in all three dimensions to fit the cladding. When cleaved (d) the QD relaxes outwards.

Some critical steps need to be followed to obtain an accurate result during the FE simulation. First, a large enough box (of cladding material) should be placed around the simulated structure to eliminate non-physical interactions from the boundaries. Second, appropriate boundary conditions should be applied to avoid any error in the simulation. The front side (which will relax outwards) should be set free, the backside is fixed, and the other four surfaces have symmetric or periodic boundary conditions: so, their movement is restricted within their own plane. Third, the initial strain matrix is applied which depends on the shape of the earlier discussed nanostructures (the initial strain matrix varies from QW to QD). In a multi-layered system with various materials, the grown layers are always strained to the substrate material to match the lattice constants. Fourth, a fine enough mesh should be generated to have high accuracy in the simulation. Finally, COMSOL calculates the equilibrium position and the final strain values. The vertical displacement component of the simulation can be compared with the outward relaxation of the cleaved surface. The local lattice constant $a_{calc}(z)$ is calculated from the final strain $\epsilon_{zz,calc}$ and initial lattice constant $a_{ini}(z)$ as follows:

$$a_{calc}(z) = \left(\epsilon_{zz,\,calc} + 1\right) \times a_{ini}(z) \qquad \textit{Equation 7}$$

which can then be compared to the local lattice constant measurements obtained from X-STM experiments.

The main steps for creating a FEM simulation on a cleaved QW/QD are as follows: create a geometry, apply the correct boundary conditions, input the initial strain conditions, and render the mesh. The geometry and mesh can be seen in Figure S3. Extra quantum dots were added behind the surface QD to simulate the high density. These QDs have

a truncated pyramid shape and the cleaving is parallel to the diagonal of the square base pyramid. As mentioned in the manuscript most of the GaAs interlayer was consumed during the dot formation and so we used 1 monolayer (ML) of GaAs as a wetting layer. This wetting layer is, together with the QDs, highlighted in Figure S3. The materials above and below the QDs are the same as mentioned in the manuscript (GaP, 20 nm AlP, 2 nm GaP, 1 ML GaAs, 3 nm QDs, 6 nm GaP:Sb, and GaP capping) for a total geometry height of 60 nm. The depth (y-direction) is large enough to avoid boundary effects (50 nm). The width (x-direction) is only 11 nm wide, which is shorter than the QD base length of 12 nm. The reason being that the two planes in the x-direction have symmetric boundary conditions which make the system act as if it were repeated infinitely along the x-direction. This was done to simulate the extremely high density of these QDs (4×10^{11} cm^{-2}). The top and bottom planes (z-direction) were also made with symmetric conditions to negate boundary effects. The backside (negative y-direction) is fixed, and the front side (positive y-direction) is free, so the surface can relax outward in that direction when "cleaved". For the relaxation to take place an initial strain is needed. All the grown layers have the QW initial strain condition (Equation 2) except the top volume of the QD which has a QD initial strain condition (Equation 3). The program can now calculate the equilibrium positions for the relaxation of the surface and the final strain values under stationary boundary conditions. The initial lattice constant of uncleaved and unstrained QD is calculated via Vegard's law using linear interpolation. The lattice constant was calculated according to Equation 7.

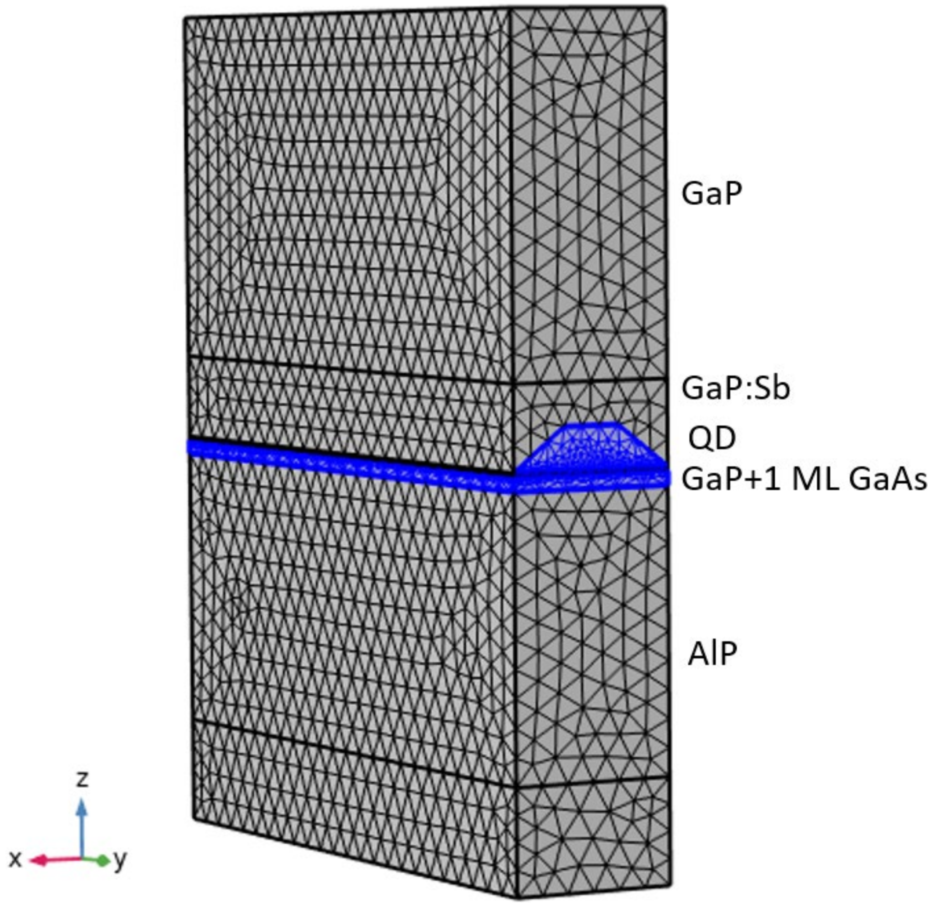

Figure S3: Geometry and mesh of the COMSOL model used to simulate the Fig:2 in the manuscript. Here, z is the growth direction and the positive y direction is normal to the cleaving plane. The most important regions highlighted in blue are 2 nm GaP and 1 monolayer (ML) GaAs.

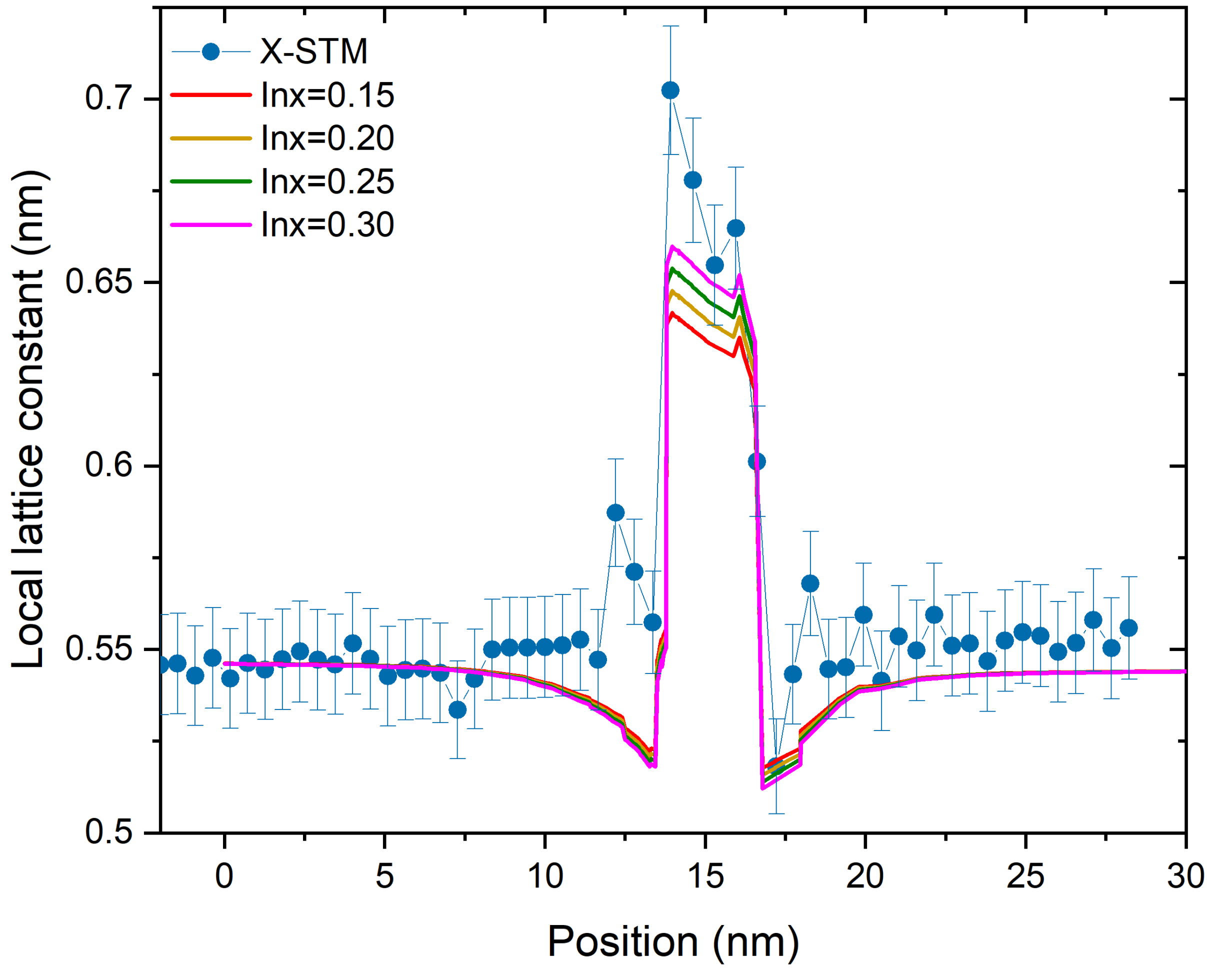

Figure S4: X-STM measured lattice constant profile with FE simulations for a QD composition with fixed As=0.85 and Sb=0.15 and varying indium concentration Inx=0.15 to 0.30, showing the effect of increasing indium concentration on lattice constant fitting. The growth direction is from the left to the right.

S-2: Effect of bias voltage on outward relaxation

The brightness in a typical filled X-STM image has two origins. One being the outward relaxation of the surface after cleavage and (or) due to the electronic effects. Typically, high negative bias (V_b) voltages were used during the measurement to suppress the electronic contribution and to extract true surface relaxation. This approximation was quite successful for direct semiconductor systems[3–5]. However, in our sample, we have tip tunneling into GaP and AlP (indirect bandgap semiconductor) and the direct bandgap QDs i.e. (InGa)(AsSb), this transition from indirect to direct bandgap material could affect the tunneling conditions.

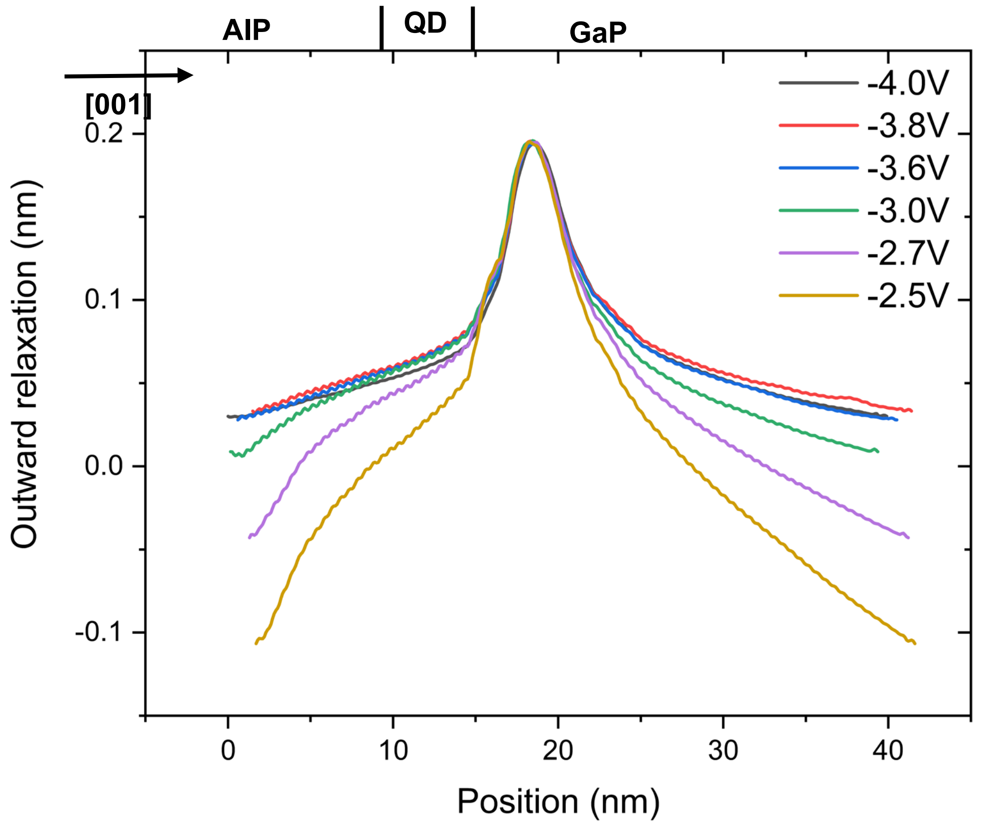

Figure S5: Effect of bias voltage on outward relaxation as a function of position. The arrow indicates the growth direction[001].

In Figure S5, the outward relaxation of a single QD measured at different bias voltages is given as a function of position in the growth direction. The shape of the relaxation profile within the QD is quite similar at all the voltages. At higher bias voltages like -3.6 V to -4.0 V, the electronic contribution to the outward relaxation was largely suppressed. However, at lower voltages (like -2.5 V to -3 V) there is a strong contribution from the electronic effects. The shape of the profile is completely different in the two regions. Tunneling into the indirect bandgap materials (GaP and AlP); the relaxation changed significantly with the bias voltage. Tunneling into the direct bandgap material (QDs); there is no notable difference in the relaxation with the measuring voltage.

S-3: Atom probe tomography

Additional information obtained from atom probe tomography (APT) measurement is provided in this section:

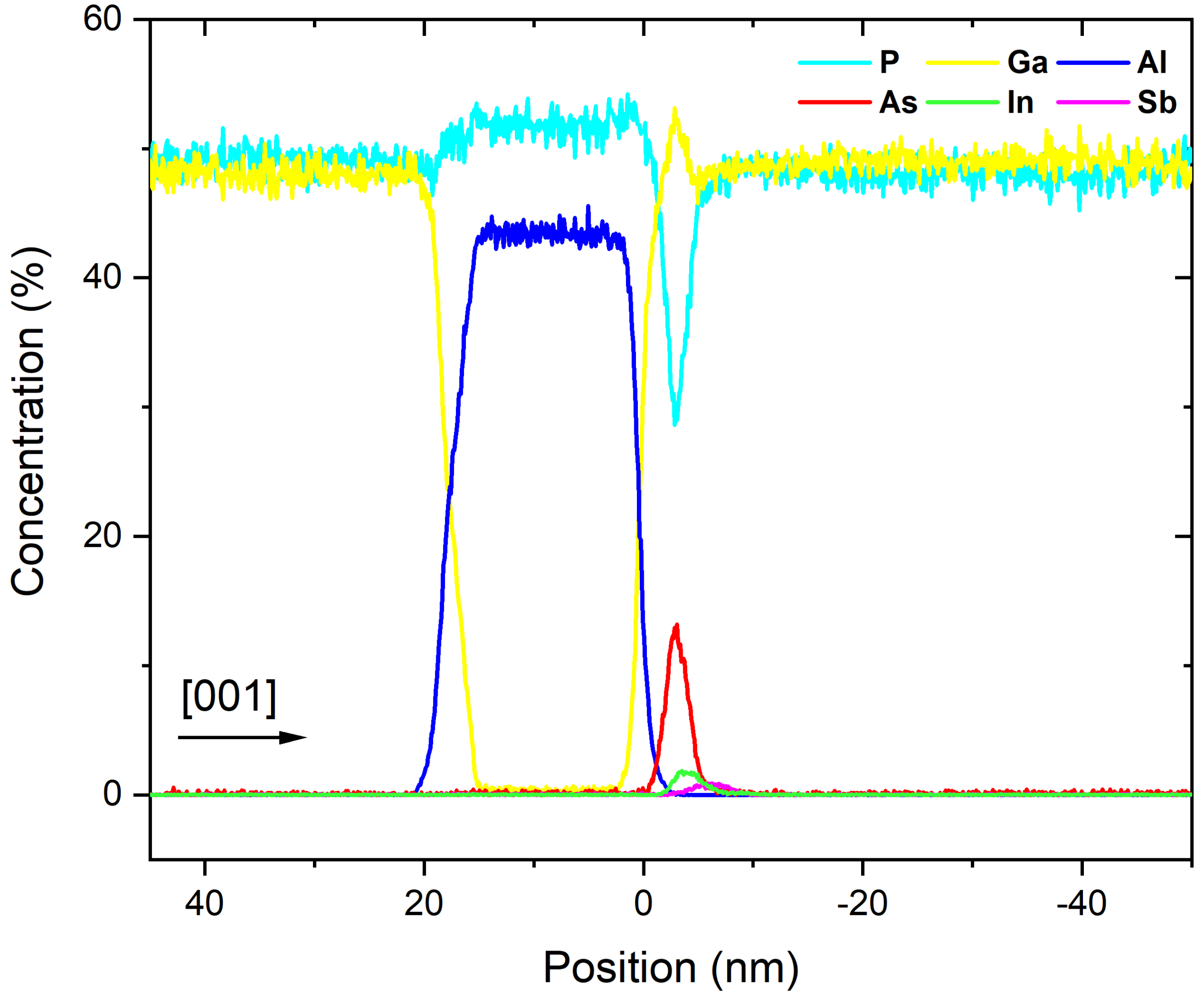

Figure S6: 1D APT concentration profile obtained from an iso-concentration surface of 15% Al (labeled as 0 nm) using a proximity histogram[6] method are shown for all the constituent elements (P, Ga, Al, As, In, and Sb). The apparent non-stoichiometry of the AlP layer is due to the unresolvable identification issue of complex phosphorus species in the 31 Da peak, as described in the manuscript. The arrow indicates the growth direction [001].

S-4: Height versus Base length of QDs

The height versus base length of QDs is reported in Fig. 2 of the manuscript (also shown below in Figure S7(a)) has a linear dependence and saturated at a height of 3.0-3.5 nm, typical for cleaved truncated pyramidal QDs. In an ideal case where all the QDs are uniform in size and shape, the height vs base length should fall on a straight line (for example the red line) but the size and shape inhomogeneities are quite common in Stranski-Krastanov QDs. The deviation of the measured height (blue points) from the linear fit (red line) represents the inhomogeneities in QDs morphology. Another reason for this deviation could be the inaccuracy in the measurement and analysis caused by the thermal drift of the STM tip.

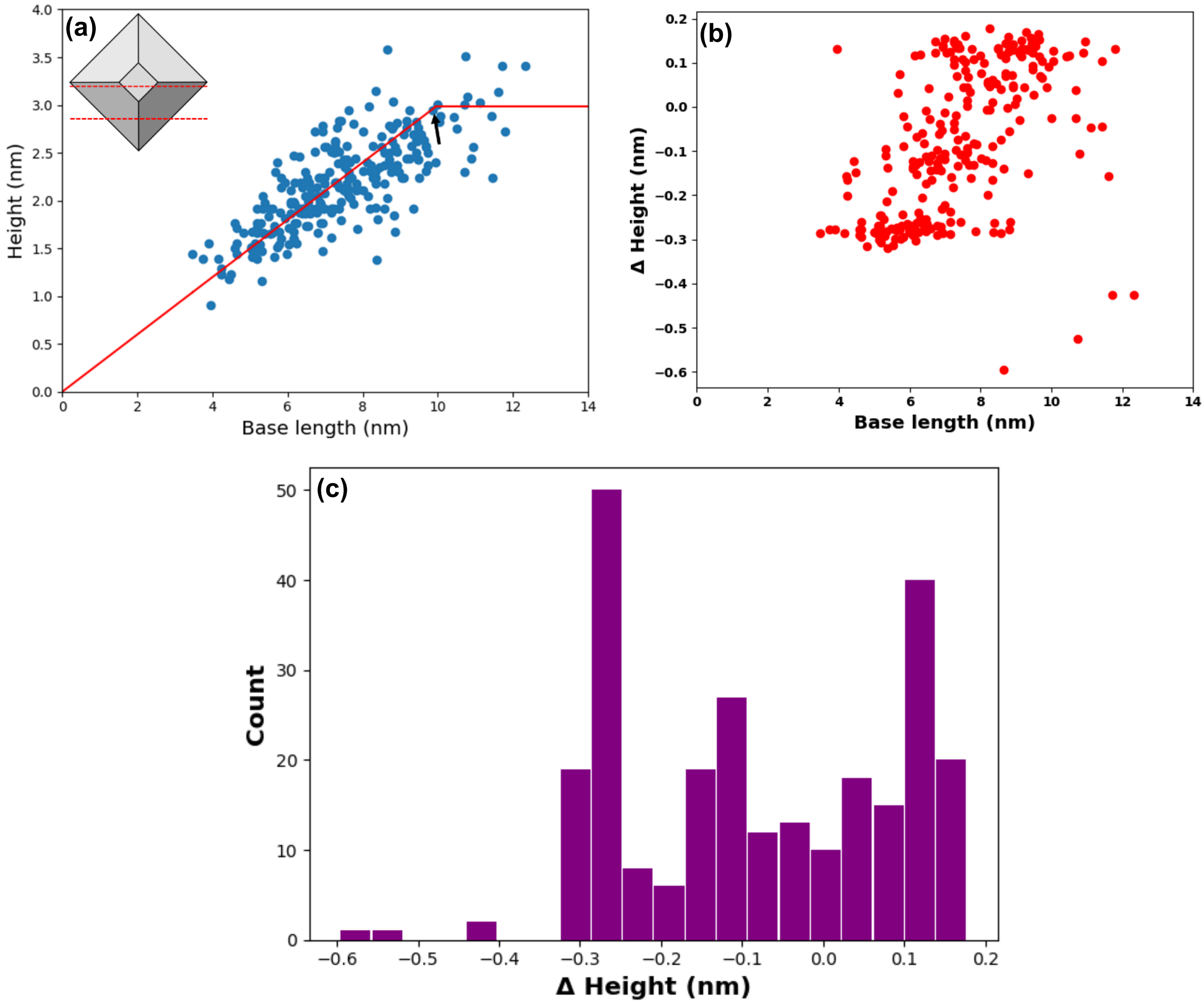

*Figure S7: **(a)** The height vs base length of the 261 QDs measured by X-STM (blue) with a linear fit (red); **(b)** The height difference (ΔH) between the linear fit and the actual measured value from X-STM; **(c)** The frequency histogram of ΔH*

In Figure S7(b), the height difference (ΔH) between the linear fit and the actual measured value from X-STM is plotted against the base length of the QDs. The maximum deviation from the linear fit is close to ±0.5 nm, this could arise either from the inhomogeneities in QDs morphology and (or) also from the inaccuracy in X-STM analysis as mentioned earlier, and Figure S7(c) represents the frequency histogram of ΔH. Overall, this analysis suggests that the error (±0.5 nm) is in the acceptable range and supports our assumption that the QDs are uniform with minimal inhomogeneities in size and shape.

References:

1. Davies, J. H., Bruls, D. M., Vugs, J. W. A. M. & Koenraad, P. M. Relaxation of a strained quantum well at a cleaved surface. *J. Appl. Phys.* **91**, 4171–4176 (2002).

2. Davies, J. H., Offermans, P. & Koenraad, P. M. Relaxation of a strained quantum well at a cleaved surface. Part II: Effect of cubic symmetry. *J. Appl. Phys.* **98**, 053504 (2005).

3. Offermans, P. *et al.* Formation of InAs quantum dots and wetting layers in GaAs and AlAs analyzed by cross-sectional scanning tunneling microscopy. *Phys. E Low-dimensional Syst. Nanostructures* **26**, 236–240 (2005).

4. Offermans, P., Koenraad, P. M., Nötzel, R., Wolter, J. H. & Pierz, K. Formation of InAs wetting layers studied by cross-sectional scanning tunneling microscopy. *Appl. Phys. Lett.* **87**, 111903 (2005).

5. Offermans, P. *et al.* Atomic-scale structure and photoluminescence of InAs quantum dots in GaAs and AlAs. *Phys. Rev. B* **72**, 165332 (2005).

6. Hellman, O. C., Vandenbroucke, J. A., Rüsing, J., Isheim, D. & Seidman, D. N. Analysis of Three-dimensional Atom-probe Data by the Proximity Histogram. *Microsc. Microanal.* **6**, 437–444 (2000).
